# Ontogenic Appearance of MHC Class I (B-F) Antigens During Chicken Embryogenesis

**DOI:** 10.1155/1990/75158

**Published:** 1990

**Authors:** D. Dunon, J. Salomonsen, K. Skjødt, J. Kaufman, B. A. Imhof

**Affiliations:** 1 Basel Institute for Immunology Grenzacherstrasse 487 Basel CH-4058 Switzerland; 2 Unstitute for Experimental Immunology University of Copenhagen Nørre Alle 71 Copenhagen ∅ DK-2100 Denmark

## Abstract

Expression of chicken MHC class (B-F) antigens during ontogeny was determined by binding of anticlass antibody and appearance of B-F transcripts by Northern blotting in chicken organs during embryogenesis until 2 weeks after hatching. MHC class transcripts first become detectable in day 6.5 of embryogenesis. B-F cell-surface expression first becomes detectable in hemopoietic organs by day 10‑12 of embryogenesis and somewhat later in nonhemopoietic organs. Flow cytometry analysis of hemopoietic cells throughout embryogenesis revealed B-Fhi and B-F1 cell populations. The percentage of B-F cells in spleen and bone marrow decreased around hatching, which could reflect either cell flows in these organs during this period or the sensitivity of hemopoietic cells to hatching stress.


*Developmental Immunology*, 1990, Vol. 1, pp. 127–135
Reprints available directly from the publisher
Photocopying permitted by license only

© 1990 Harwood Academic Publishers GmbH
Printed in the United Kingdom

### Ontogenic Appearance of MHC Class I (B-F) Antigens During Chicken Embryogenesis

D. DUNON,*† J. SALOMONSEN,‡ K. SKJØDT,‡ J. KAUFMAN,† and B. A. IMHOF†

†*Basel Institute for Immunology, Grenzacherstrasse 487, CH-4058 Basel, Switzerland*
‡*Institute for Experimental Immunology, University of Copenhagen, Nørre Alle 71, DK-2100 Copenhagen Ø, Denmark*

Expression of chicken MHC class I (B-F) antigens during ontogeny was determined by binding of anticlass I antibody and appearance of B-F transcripts by Northern blotting in chicken organs during embryogenesis until 2 weeks after hatching. MHC class I transcripts first become detectable in day 6.5 of embryogenesis. B-F cell-surface expression first becomes detectable in hemopoietic organs by day 10–12 of embryogenesis and somewhat later in nonhemopoietic organs. Flow cytometry analysis of hemopoietic cells throughout embryogenesis revealed B-F$^{hi}$ and B-F$^{lo}$ cell populations. The percentage of B-F$^{+}$ cells in spleen and bone marrow decreased around hatching, which could reflect either cell flows in these organs during this period or the sensitivity of hemopoietic cells to hatching stress.

KEYWORDS: MHC class I, B-F antigens, chicken, embryos.

### INTRODUCTION

Classical MHC class I antigens are highly polymorphic membrane glycoproteins that are expressed by most adult somatic cells in vertebrates and are essential in T-cell immunity (Hood et al., 1983). They consist of an $\alpha$-chain glycoprotein (approximately 45 kD) noncovalently associated with $\beta_2$-microglobulin (12 kD). In mouse, the fertilized oocytes are MHC class I positive (probably maternally derived; Palm et al., 1971; Sawicki et al., 1981) and become negative by the cleavage stage of the embryo (Heyner et al., 1980). Expression of MHC class I genes at the level of both mRNA and surface protein becomes detectable again only after the midsomite stage of embryogenesis and remains very low throughout gestation (Ozato et al., 1985). MHC class I expression does not begin simultaneously throughout the embryo, since lung, skin, and limb buds become MHC class I positive after 11 days of gestation, whereas gut, kidney, and gonads remain negative (Kirkwood and Billington, 1981).

The biochemical structure, adult tissue distribution, and functional attributes of B-F antigens show that they are the chicken equivalents of the mammalian MHC class I molecules (Guillemot et al., 1989). Our knowledge of the timing and mechanisms of chicken MHC class I antigen expression during embryogenesis is incomplete. In contrast to murine MHC class I expression, B-F antigens were not detected in previous studies during embryonic development except in thymus. The first B-F positive cells detected by flow cytometry were thymocytes from 18-day-old embryos, whereas bursal cells, peripheral blood leucocytes, and red blood cells expressing B-F antigen were not detectable until a few days after hatching (day 21) (Sgonc et al., 1987).

This issue warrants further examination because the class I antigens appear essential for the development of cytotoxic T-cell immune repertoire during ontogeny. In the present work, we examine the expression of B-F mRNA and reexamine surface antigens in embryos. We found that B-F antigens are expressed by day 10–11 of embryogenesis. In addition, the size of chicken embryos allowed us to analyze B-F expression in hemopoietic organs by flow cytometry. The percentage of B-F$^{+}$ cells in thymus and bursa increases steadily during the development in contrast to the bone marrow and the spleen, where a great decrease is observed around hatching.

*Corresponding author.

### RESULTS

### Analysis of MHC Class I Heavy-Chain Transcripts During Ontogeny

RNA was prepared from organs isolated from embryos with the aid of microdissection instruments. By day 10 of embryogenesis, there were sufficient cells in the various organs to obtain enough RNA for analysis. When these RNAs were hybridized with an MHC class I heavy-chain probe recognizing all known B-F genes (Kaufman et al., 1989; Skjoedt et al., submitted), two transcripts of 1.5 and 1.95 kb (Fig. 1) were detected. Most of the analyzed organs expressed these transcripts by day 10–13 of embryogenesis (Fig. 1 and Table 1). Moreover, a very low level of B-F transcripts could be detected in total RNA preparation from 6.5- and 8.5-day-old embryos (data not shown). During development, the amounts of the two transcripts were equivalent in the different organs except for the brain, the kidney, and the liver. In these organs, the 1.5-kb transcript is the major one.

TABLE 1
B-F Transcripts During Chicken Embryogenesis[a]

| | 1.5-kb transcript | | | | 1.95-kb transcript | | | |
|---|---|---|---|---|---|---|---|---|
| Days of embryogenesis | 10 | 13 | 17 | 2W | 10 | 13 | 17 | 2W |
| Brain | + | + | + | +++ | − | − | − | ++ |
| Kidney | + | + | ++ | ++++ | − | − | + | ++++ |
| Liver | + | ++ | ++++ | +++++ | + | + | ++ | +++++ |
| Intestine | − | + | ++ | +++++ | − | + | ++ | +++++ |
| Blood cells | + | + | + | ND | + | + | + | ND |
| Spleen | − | + | ++ | +++ | − | + | ++ | +++ |
| Bursa | − | ++ | +++ | ++++ | − | ++ | +++ | ++++ |
| Thymus | + | ++ | ++ | ++++ | + | + | ++ | ++++ |

[a]The number of (+) corresponds approximately to the amount of transcript; (−) indicates that the transcript was not detected; however, in most negative samples, very long exposures of the blots showed very low amounts of transcripts. ND, not determined. Numbers indicate the time of incubation of eggs in days, and 2W corresponds to 2-week-old chickens.

### Appearance of the B-F Antigen During Chicken Embryogenesis

In order to determine when and where MHC class I was expressed, we stained frozen sections of embryonic tissues with the monoclonal antibody F21-2 (Crone and Simonsen, 1987; Salomonsen et al., 1987), which recognizes a monomorphic chicken MHC class I $\alpha$-chain epitope and thus presumably detects all the different B-F antigens. This analysis shows that B-F antigen expression occurs in all organs examined during embryogenesis except for the brain (Fig. 2 and Table 2).

The thymus is the first organ that expresses detectable B-F antigen. Rare B-F$^+$ single cells were observed by immunofluorescence before day 10 of embryogenesis (not shown). This expression

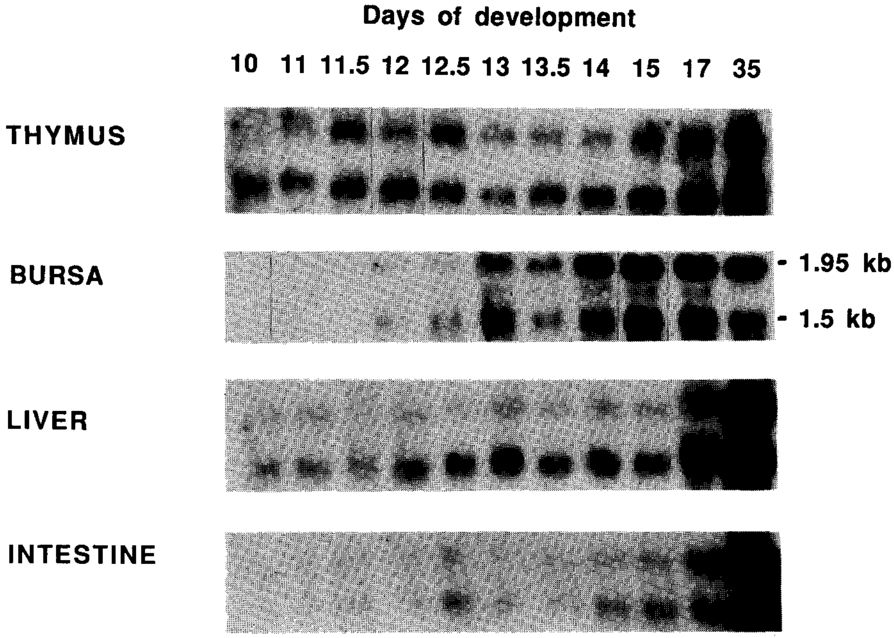

FIGURE 1. MHC class I $\alpha$-chain transcripts during embryogenesis. Northern blots were performed using around 5 $\mu$g total RNA from H.B21 embryonic tissues (thymus, bursa, liver, and intestine), standardized using hybridization of $\beta$ actin cDNA. Numbers above each lane indicate the age of embryos. These Northern blots were hybridized with B-F19 cDNA. The size of the transcripts are 1.5 and 1.95 kb.

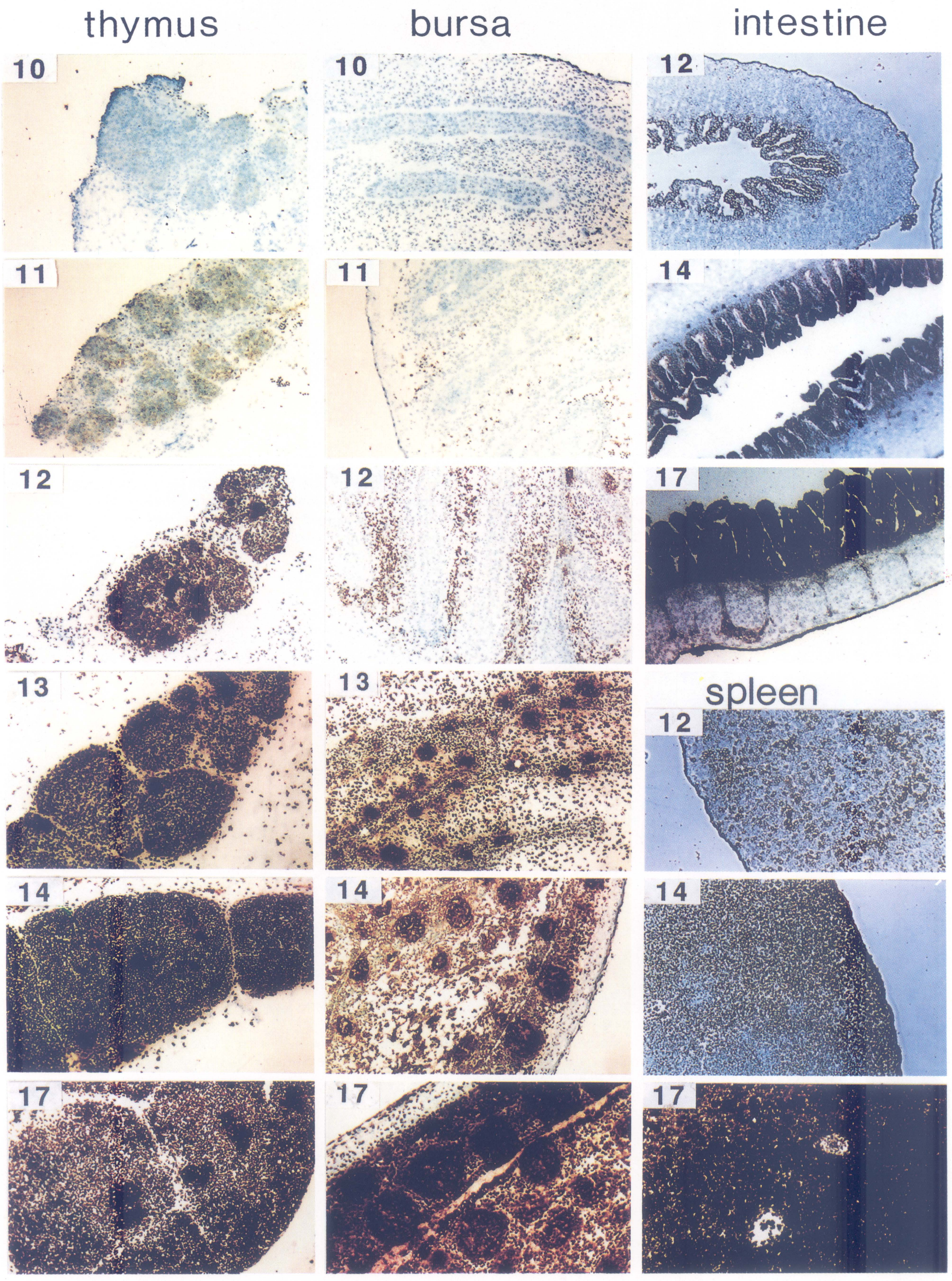

FIGURE 2. Immunostaining of B-F antigens in embryonic organs. Thymus, bursa, intestine, and spleen were taken from H.B21 chicken embryos of different stages. Numbers indicate the time of incubation of eggs in days. Frozen sections were immunostained by antibody F21-2 and detected by a peroxidase-coupled antimouse IgG antibody. Controls were stained with the peroxidase-conjugated antibody only. Magnification 1000×. (See Colour Plate VI at the back of this publication.)

TABLE 2
B-F Antigen Expression During Ontogeny of the Chicken[a]

| Days of development | 10 | 11 | 12 | 13 | 14 | 15 | 17 | 1W |
|---|---|---|---|---|---|---|---|---|
| Brain | | | | − | | − | − | ++ |
| Kidney | − | − | − | ± | ± | + | ++ | +++ |
| Liver | − | − | − | + | ++ | ++ | ++ | +++ |
| Intestine | − | − | ± | + | ++ | +++ | ++++ | ++++ |
| Spleen | − | − | ± | + | ++ | +++ | ++++ | ++++ |
| Bone marrow | − | − | ± | ++ | ++ | ++ | ++++ | ++++ |
| Bursa | − | + | + | ++ | +++ | +++ | ++++ | ++++ |
| Thymus | ± | + | + | +++ | ++++ | ++++ | ++++ | ++++ |

[a]The number of (+) corresponds approximately to the amount of B-F antigen detected on stained frozen sections; (−) indicates that B-F antigen was not detected. Numbers indicate the time of incubation of eggs in days, and 1W corresponds to 1-week-old chickens. Data for bone marrow are based on FACScan analysis. Rare B-F$^+$ single cells were detected in the thymus before day 10.

increases steadily during embryogenesis and the medullary thymocytes of 17-day-old embryos become strongly B-F positive as reported for adult thymuses (Pink et al., 1985).

The first positive bursal cells, expressing low levels of MHC class I antigens, were detected in the mesenchyme at day 11 of embryogenesis. Then highly B-F positive cells appeared in the bursal follicles (day 13). These results suggest that B-cell precursors colonizing the bursa were B-F positive. In fact, hemopoietic pluripotent stem cells and committed progenitors in murine adult bone marrow bear surface class I antigens (Russel and Van den Hengh, 1979; Fitchen et al., 1982).

B-F positive cells appeared on the spleen and on the intestine epithelium at day 12 and the labeling increased steadily during embryogenesis. At day 17, a strong staining was observed in intestine on the villi and blood vessels, but little or low labeling was detected on the *muscularis.*

### Quantitative Analysis of MHC Class I Positive Cells in Hemopoietic Organs

The total cells of hemopoietic organs were analyzed by flow cytometry starting at day 8 of embryogenesis. This study revealed two B-F positive splenocyte populations, named B-F$^{lo}$ and B-F$^{hi}$, during chicken embryogenesis (Figs. 2 and 3). The B-F$^{hi}$ cell population appeared in 11.5-day-old spleen and increased steadily throughout embryogenesis. Interestingly, this population almost disappeared around hatching (day 21) before becoming the major splenocyte population in adult chicken. Double labeling experiments were used to analyze the cell types involved (Table 3). At day 16 of embryogenesis, 74% of B-F$^{hi}$ cells are MHC class II positive and 43% of this population expressed a surface marker for the myeloid lineage recognized by antibody 51-2 (Kornfeld et al., 1983). A low percentage (13%) of the B-F$^{hi}$ cells are probably of the B lymphoid lineage (defined by the Bu-1 marker, which is found on cells of the B-cell lineage and a subset of macrophages; Houssaint et al., 1987). In contrast, at day 28 (1 week after hatching), the B-F$^{hi}$ cell population contains only 17% of myeloid lineage cells but 54% of lymphoid lineage cells (calculated from Bu-1 or CD2 positive cells in Table 3). Thus, these results show that between day 16 of embryogenesis and 1 week after hatching (day 28), the B-F$^{hi}$ cell population in spleen undergoes an important shift from myeloid to lymphoid lineage cells. In addition, the maximum percentage of B-F positive cells (high and low) occurred around day 17–18 of embryogenesis, the hatching appearing correlated with a fall of class I MHC antigen expression on splenocytes (Fig. 4). The same decrease is observed in bone marrow.

Only the B-F$^{lo}$ cell population was observed in embryonic thymus. On the contrary, only the B-F$^{hi}$ cell population was observed in embryonic bursa (not shown).

In agreement with the staining of frozen sections, B-F positive cells appeared in bursa, thymus, spleen, and bone marrow between day 10 and 12 of embryogenesis (Fig. 4). The fastest kinetics of appearance of B-F positive cells is observed for thymus since almost all thymocytes were B-F positive at day 13 of development. In accordance with earlier data (Sgonc et al., 1987), we found that peripheral erythrocytes were B-F negative during embryogenesis, MHC class I expression increasing steadily after hatching.

TABLE 3
Analysis of B-F$^{hi}$ Splenocyte Population During Chicken Development[a]

| Days of development | 16 | 21 | 23 | 28 |
|---|---|---|---|---|
| B-F$^{hi}$ | 38 | 10 | 10 | 35 |
| B-F$^{hi}$ Bu-1$^+$ | 5 | 2 | 0.5 | 10 |
| B-F$^{hi}$ B-L$^+$ | 28 | 6 | 6 | 11 |
| B-F$^{hi}$ B-L$^+$ 51.2$^+$ | 12 | 6 | 4 | 6 |
| B-F$^{hi}$ CD2$^+$ | <0.5 | <0.5 | <0.5 | 9 |

[a]Data are expressed in percentage of total splenocytes. Antibody 51.2 recognizes a marker present on the cell surface of myeloid lineage cells (Kornfeld et al., 1983).

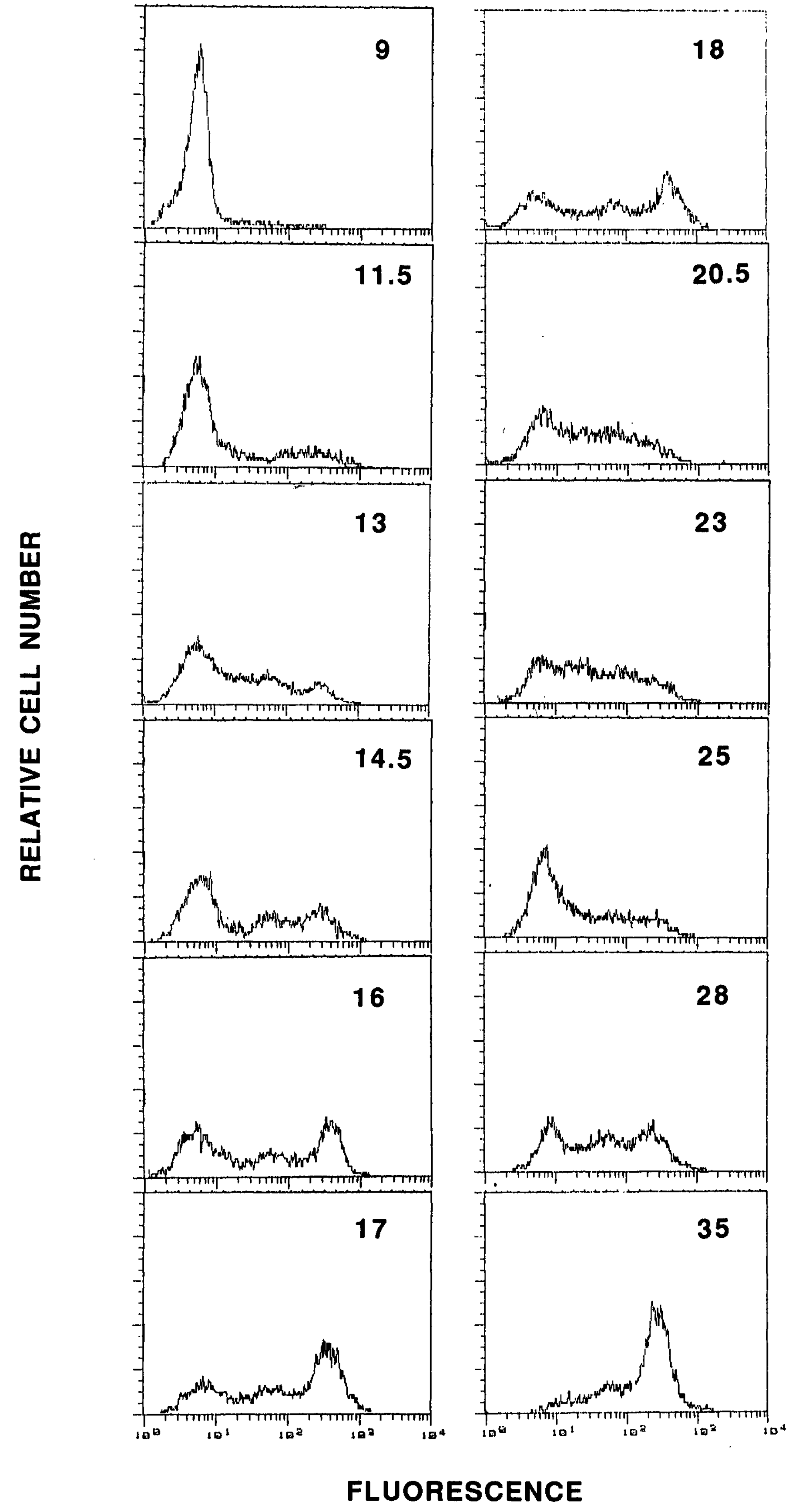

FIGURE 3. Analysis of B-F positive splenocytes during development of chicken. Total splenocytes from H.B21 embryos were collected and analyzed by flow cytometry for B-F$^+$ cells using the antibody F21-2. Numbers indicate the age of development in days. Controls were done at every stage with the second antibody only and are identical to the profile obtained for day 9 of embryogenesis. Two B-F positive cell populations, B-F$^{hi}$ and B-F$^{lo}$, are observed during ontogeny.

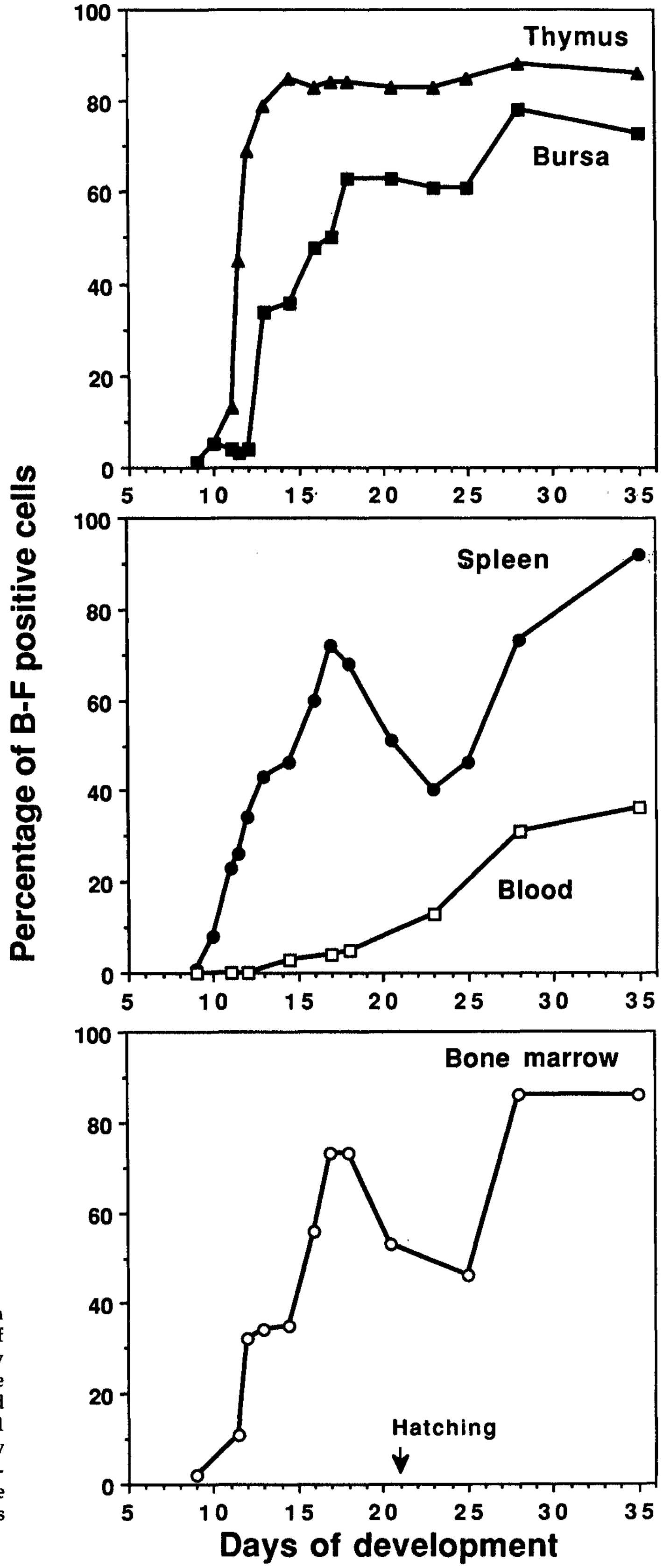

FIGURE 4. Ontogenic appearance of B-F positive cells in various hemopoietic tissues. Percentage of B-F$^+$ cells of different hemopoietic organs were determined by flow cytometry using the antibody F21-2. This percentage includes both the B-F$^{hi}$ and B-F$^{lo}$ cell populations detected in Fig. 3. These curves have been established for the H.B21 chicken strain. These results have been checked for few stages in H.B19 and CB chicken strains. Minor discrepancies observed between Table 2, Fig. 2, and Fig. 4 are due to the difference of sensitivity of FACScan analysis and staining of frozen sections.

### DISCUSSION

We report here that B-F mRNA is detectable before day 10 of embryogenesis and that, accordingly, surface class I antigens are detectable from day 10 onwards. Furthermore, we found that the level of B-F mRNA remains lower than that in adult tissues (Fig. 1), but both the level of B-F mRNA and B-F cell-surface expression increases during embryogenesis (Figs. 2 and 4). This work was performed using H.B21 chicken strain, but B-F expression was also checked at several stages with CB and H.B19 chicken embryos.

The relatively early appearance of B-F antigen found in this study is in contrast to previous reports in which antigen expression was not detected until day 17–18 of embryogenesis (Sgonc et al., 1987; Pink et al., 1985). This discrepancy may be due to the use of lower-affinity antibodies and less efficient assays. Indeed, the silver enhancement method used in this report is highly sensitive. On the other hand, the present results are in agreement with those observed during mouse embryogenesis (Ozato et al., 1985). At the moment, we cannot rule out that antibody F21.2 and/or B-F19 cDNA probe also detect nonpolymorphic MHC class I molecules such as Qa and Tla antigens described in other species.

The B-F19 cDNA hybridized to two transcripts of 1.5 and 1.95 kb in all studied organs of H.B21 chickens. These two transcripts have already been observed in CB chicken liver, thymus, and spleen; however, the longer transcript is the only one detectable in chicken B- and T-cell lines (Guillemot et al., 1988). The relative amounts of these two transcripts in each organ of H.B21 embryos are stable during embryogenesis, but differ between organs. Brain, kidney, and liver contain larger amounts of 1.5-kb transcript than of 1.95-kb transcript, whereas the other organs contain equivalent amounts of the two transcripts.

Interestingly, $\beta_2$-microglobulin molecule and transcripts appear first in thymus. In contrast to MHC class I heavy chain, a peak of free $\beta_2$-microglobulin synthesis occurs between day 12 and 14 of embryogenesis (Dunon et al., 1990). Double immunostaining of thymus sections showed that a large subset of free $\beta_2$-microglobulin positive cells was MHC class I heavy-chain negative. It has been suggested that $\beta_2$-microglobulin is secreted as a free molecule during this period, which corresponds to the second wave of thymus colonization. Free $\beta_2$-microglobulin is chemotactic for pre-T cells and thus may be involved in thymus homing of hemopoietic precursors (Dunon et al., 1990).

Cell-surface expression of most mammalian class I heavy chain is dependent on their noncovalent binding to $\beta_2$-microglobulin during biosynthesis (Ploegh et al., 1981). As previously observed in mouse embryos (Morello et al., 1985), appearance of $\beta_2$-microglobulin transcripts in liver during chicken embryogenesis (Dunon et al., 1990) clearly occurs later than that of B-F transcripts. Thus, the failure of B-F antigen expression during early embryogenesis, at least in this organ, could be due to the absence of $\beta_2$-microglobulin (as already suggested for tadpole cells of *Xenopus;* Flajnik et al., 1986). This hypothesis could be extended to brain, kidney, and intestine.

The presence of two B-F positive cell populations (B-F$^{hi}$ and B-F$^{lo}$) in bone marrow and spleen and the large decrease of one of these populations a few days before hatching could reflect the cell flows and/or the expansion of particular cell populations occurring in these organs during this period. In spleen, emigration of granulocytes occurs within 3 or 4 days around hatching and lymphocyte homing starts just before hatching (Yassine et al., 1989), which is in agreement with our double-staining experiments. In this organ, the decrease of B-F expression could reflect this transition. Thus, the B-F$^{hi}$ cell population observed around day 17–18 of embryogenesis corresponds essentially to granulocytes and those observed one week after hatching to lymphocytes. The decrease of B-F positive cells in bone marrow could be due to an increase of hemopoiesis just before hatching, most blood cells being class I negative at this stage (Fig. 4). Alternatively, the decrease of B-F positive hemopoietic cells in bone marrow and spleen could be a consequence of hatching stress, since, for instance, in mammals, lymphoid cells are sensitive to steroid hormones or PGE1 (Whitlock and Witte, 1982; Hayashi et al., 1984; Deugnier et al., 1989).

### MATERIALS AND METHODS

### Animals

Embryos of White Leghorn MHC homozygous chicken strains CB (*B12* haplotype), H.B19, and H.B21 were from animals kept at Gipf-Oberfrick (Switzerland). Fertilized eggs were incubated at 38°C and 80% humidity in a ventilated incubator (Pink et al., 1985). The length of the third toe was used as the criterion to stage embryos (Dunon et al., 1990).

### Organs

Organs of precisely staged embryos (H.B21) were taken by microdissection and collected on ice. As a source of RNA, organs from 10–12 embryos were pooled and stored at −80°C. For frozen sections, organs were immediately embedded in Tissue-Tek (ImmunoBiologicals) without fixation and slowly frozen on dry ice. Blocks could be stored at −70°C for several weeks.

### Cells

Embryonic bone marrow cells were flushed from the cavity of isolated femur and tibia using a 25G 5/8-inch-long syringe containing DMEM, buffered with 20 mM HEPES, pH 7.55, without sodium bicarbonate (Imhof et al., 1988). A single-cell suspension was made by pipetting followed by filtration through a nylon sieve (mesh width of 25 μm; Nytal P-25 my, SST, Thal, Switzerland). Several embryonic thymic lobes, bursa, or spleen were carefully pressed between two nylon sieves (mesh width of 100 μm; VS-Monodur PA-100, SST, Thal, Switzerland) and cells were mechanically released by vigorous pipetting.

Total cells were analyzed for presence of MHC class I antigen by flow cytometry using a FACScan (Becton Dickinson) after staining with the F21-2 antibody followed by FITC-labeled antimouse Ig (Silenus). Alternatively, bone marrow cells were diluted five times with PBS at room temperature and centrifuged (60 g, 15 min, room temperature). The upper two-thirds of the plasmaphase was ficolled (200 g, 15 min, room temperature), which resulted in an enrichment of leucocytes. These cells were used in double-staining experiments.

### Northern Blots

Total RNA from organs was prepared by a modified guanidinium/CsCl centrifugation method (MacDonald et al., 1987). Tissues were homogenized in ten volumes of 4 M guanidinium thiocyanate, 0.05 M sodium acetate, 2 mM EDTA, and 1 M β-mercaptoethanol. Solid CsCl was added to reach a concentration of 4.5 M. Homogenates were layered onto a cushion of 6.7 M CsCl (d = 1.82 g/ml), 0.05 M sodium acetate, and 1 mM EDTA. After overnight centrifugation at 40,000 rpm and 20°C in a 70.1 Ti rotor (Beckman), RNA appeared as a band in the CsCl gradient and was recovered with a syringe and ethanol-precipitated.

About 5 μg of total RNA from the different organs were heated at 60°C in 50% formamide, 4-morpholipropane sulfonic acid buffer (pH7; 0.2 M MOPS, 50 mM sodium acetate, and 10 mM EDTA), and then electrophoresed in 1% agarose gel containing 6% formaldehyde in the running buffer. RNAs were blotted onto nylon membrane (Hybond N, Amersham). The probes used to hybridize these blots were chicken MHC class I antigen cDNA B-F19 (Kaufman et al., 1989; Skjoedt et al., submitted) and mouse β-actin cDNA (pAL41β, Alonso et al., 1986). These probes were labeled by random priming (random priming kit, Boehringer) with $^{32}$PdCTP. Filters were hybridized with the probe at 42°C in 50% formamide, 5×SSPE, 2×Denhardt (0.2% polyvinylpyrrolidone/0.2% Ficoll/0.2% bovine serum albumin), 5% dextran sulfate, and 100 μg of salmon sperm DNA per ml. Blots were washed in 0.1×SSC, 0.1% SDS at 55°C, and they were further exposed to Kodak X-OMAT film for 1–3 days.

### Immunolabeling

The mouse monoclonal antibody F21-2 recognizes a monomorphic chicken MHC class I α-chain epitope (Crone and Simonsen, 1987; Salomonsen et al., 1987). Antibody 51-2 recognizes a marker present on the cell surface of myeloid lineage cells (Kornfeld et al., 1983), antibody 2G11 recognizes MHC class II antigens (Kaufman et al., 1990), antibody 2-4-8 recognizes CD2 antigen (Vainio et al., submitted), and antibodies L-22 and 11-G-2 recognize Bu-1a and Bu-1b antigens, respectively (Pink et al., 1983; Veromaa et al., 1988). In double-staining experiments, some of these antibodies were coupled to biotin and detected with phycoerythrin coupled to streptavidin (Southern Biotechnology Associates, Birmingham, AL). Second antibodies were fluorescein labeled sheep antimouse Ig antibodies (Silenus, Hawthorn, Australia) or peroxidase-coupled rabbit antimouse Ig (Serotec, Oxford, UK).

Frozen sections of embryonic organs were cut to a thickness of 5 μm on a cryostat (Leitz, Wetzlar), fixed with acetone, rehydrated in phosphate-buffered saline (PBS) containing 1% bovine serum albumine (BSA). F21-2 antibody was used as undiluted hybridoma supernatant. Diaminobenzidine was used as a substrate for horse radish peroxidase and the signal was increased by using a silver amplification kit (Amersham). The tissue was counterstained by methylgreen. Photos were obtained using a CCD color camera module (XC 007, AVT Leuzinger, Basel, Switzerland) and a color video printer (UP 5000 P, Sony).

### ACKNOWLEDGMENTS

We thank Birgit Kugelberg and Barbara Hesse for technical assistance, Viktor Hasler for animal facilities, Hans Spalinger and Beatrice Pfeiffer for photography, Dr. Olli Vainio for antibody 2-4-8, Dr. Thomas Graf (EMBL, Heidelberg) for antibody 51.2 and Drs. Alexandra Livingstone and Louis Du Pasquier for critical reading of the manuscript.

The Basel Institute for Immunology was founded and is supported by F. Hoffmann-La Roche Ltd., Switzerland.

*(Received June 13, 1990)*

*(Accepted July 19, 1990)*

### REFERENCES

Alonso S., Minty A., Beurlet Y., and Buckingham M. (1986). Comparison of three actin coding sequences in the mouse. Evolutionary relationships between the actin genes of warm-blooded vertebrates. J. Mol. Evol. **23:** 11–22.

Crone M., and Simonsen M. (1987). Avian major histocompatibility complex. *In:* Avian immunology: Basis and practice, vol II, Toivanen A., and Toivanen P., Eds. (Boca Raton, FL: CRC Press), pp.25–41.

Deugnier M.A., Imhof B.A., Bauvois B., Dunon D., Denoyelle M., and Thiery J.P. (1989). Characterization of rat T cell precursors sorted by chemotactic migration toward thymotaxin. Cell **56:** 1073–1083.

Dunon D., Kaufman J., Salomonsen J., Skjoedt K., Thiery J.P., and Imhof B.A. (1990). T-cell precursor migration toward $\beta 2$-microglobulin is involved in thymus colonization of chicken embryos. EMBO J., in press.

Fitchen J.H., Hayi E.F., and Ferrone S. (1982). Expression of the H-2 antigenic complex on murine hematopoietic stem cells. Scand. J. Immunol. **15:** 547–551.

Flajnik M.F., Kaufman J.F., Hsu E., Manes M., Parisot R., and Du Pasquier L. (1986). Major histocompatibility complex encoded molecules are absent in immunologically competent *Xenopus* before metamorphosis. J. Immunol. **137:** 3891–3899.

Guillemot F., Billault A., Pourquie O., Behar G., Chausse A.-M., Zoorob R., Kreiblich G., and Auffray C. (1988). A molecular map of the chicken major histocompatibility complex: The class II $\beta$ genes are closely linked to the class I genes and the nucleolar organizer. EMBO J. **7:** 2275–2785.

Guillemot F., Kaufman J.F., Skjoedt K., and Auffray C. (1989). The major histocompatibility complex in the chicken. Trends Genet. **5:** 300–304.

Hayashi J., Medlock E.S., and Goldschneider I. (1984). A selective culture system for generating terminal deoxynucleotidyl transferase positive (TdT+) lymphoid precursor cells in vitro. I. Description of the culture system. J. Exp. Med. **160:** 1622–1639.

Heyner S., Hunziker R.D., and Zink G.L. (1980). Differential expression of minor histocompatibility antigens on the mouse oocyte and preimplantation developmental stages. J. Reproduc. Immunol. 2269–2279.

Hood L., Steinmetz M., and Malissen B. (1983). Genes of the major histocompatibility complex of the mouse. Annu. Rev. Immunol. **1:** 529–568.

Houssaint E., Diez E., and Pink J.R.L. (1987). Ontogeny and tissue distribution of the chicken Bu-1a antigen. Immunology **62:** 463–470.

Imhof B.A., Deugnier M.A., Girault J.M., Champion S., Damais C., Itoh T., and Thiery J.P. (1988) Thymotaxin: A thymic epithelial peptide chemotactic for T-cell precursors. Proc. Natl. Acad. Sci. USA **85:** 7699–7703.

Kaufman J., Salomonsen J., and Skjoedt K. (1989). B-G cDNA clones have multiple small repeats and hybridize to both chicken MHC regions. Immunogenetics **30:** 440–451.

Kaufman J., Skjoedt K., Salomonsen J., Simonsen M., Du Pasquier L., Parisot R., and Riegert P. (1990). MHC-like molecules in some nonmammalian vertebrates can be detected by some cross-reactive xenoantisera. J. Immunol. **144:** 2258–2272.

Kirkwood K.J., and Billington W.D. (1981). Expression of serologically H-2 antigens on mid-gestation mouse embryonic tissues. J. Embryol. Exp. Morph. **61:** 207–219.

Kornfeld S., Beug H., Doederlein G., and Graf T. (1983). Detection of avian hematopoietic cell surface antigens with monoclonal antibodies to myeloid cells. Exp. Cell Res. **143:** 383–394.

MacDonald R.J., Swift G.H., Przybyla A.E., and Chirgwin J.M. (1987). Isolation of RNA using guanidium salts. Methods Enzymol. **152:** 219–227.

Morello D., Duprey P., Israel A., and Babinet C. (1985). Asynchronous regulation of mouse H-2D and Beta-2 microglobulin RNA transcripts. Immunogenetics **22:** 441–452.

Ozato K., Wan Y.-J., and Orrison B.M. (1985). Mouse major histocompatibility class I gene expression begins at midsomite stage and is inducible in earlier-stage embryos by interferon. Proc. Natl. Acad. Sci. USA **82:** 2427–2431.

Palm J., Heyner S., and Brinster R.L. (1971). Differential immunofluorescence of fertilized mouse eggs with H-2 and non-H-2 antibody. J. Exp. Med. **133:** 1282–1293.

Pink J.R.L., Kieran M.W., Rijnbeek A.M., and Longenecker B.M. (1985). A monoclonal antibody against chicken MHC Class I (B-F) antigens. Immunogenetics **21:** 293–297.

Pink J.R.L., and Rijnbeek A.M. (1983). Monoclonal antibodies against chicken lymphocyte surface antigens. Hybridoma **2:** 287–296.

Ploegh H.L., Orr H.T., and Strominger J.L. (1981). Major histocompatibility antigens: The human (HLA-A, -B, -C) and murine (H-2K, H-2D) class I molecules. Cell **24:** 287–299.

Russell J.L., and Van den Engh G. (1979). The expression of histocompatibility-2 antigens on hemopoietic stem cells. Tissue Antigens **13:** 45–52.

Salomonsen J., Skjoedt K., Crone M., and Simonsen M. (1987). The chicken erythrocyte specific MHC antigen. Characterization and purification of the B-G antigen by monoclonal antibodies. Immunogenetics **25:** 373–382.

Sawicki J.A., Magnuson T., and Epstein C.J. (1981). Evidence for expression of the paternal genome in the two-cell mouse embryo. Nature **294:** 450–451.

Sgonc R., Hála K., and Wick G. (1987). Relationship between the expression of class I antigens and reactivity of chicken thymocytes. Immunogenetics **26:** 150–154.

Veromaa T., Vainio O., Eerola E., and Toivanen P. (1988). Monoclonals antibodies against chicken Bu-1a and Bu-1b alloantigens. Hybridoma **7:** 41–48.

Whitlock C.A., and Witte O.N. (1982). Long-term culture of B lymphocytes and their precursors from murine bone marrow. Proc. Natl. Acad. Sci. USA **79:** 3608–3612.

Yassine F., Fedecka-Bruner B., and Dieterlen-Lièvre F. (1989). Ontogeny of the chick embryo spleen—a cytological study. Cell Diff. Dev. **27:** 29–45.

